# *In vitro* and *in vivo* hepatoprotective effects of the total alkaloid fraction of *Hygrophila auriculata* leaves

**DOI:** 10.4103/0253-7613.64500

**Published:** 2010-04

**Authors:** Vasanth P. Raj, Raghu H. Chandrasekhar, Vijayan P., Dhanaraj S. A., Mallikarjuna C. Rao, Venkata J. Rao, K. Nitesh

**Affiliations:** Manipal College of Pharmaceutical Sciences, Manipal University, Manipal - 576 104, India; 1 J.S.S.College of Pharmacy, Manipal University, Manipal - 576 104, India

**Keywords:** HepG2, hepatocytes, hepatoprotective, *Hygrophila auriculata*, total alkaloidsIntroduction

## Abstract

**Objective:**

To investigate the total alkaloid fraction of the methanol extract of leaves of *Hygrophila auriculata* for its hepatoprotective activity against CCl4-induced toxicity in freshly isolated rat hepatocytes, HepG2 cells, and animal models.

**Materials and Methods:**

Mature leaves of *H. auriculata* were collected, authenticated, and subjected to methanolic extraction followed by isolation of total alkaloid fraction. Freshly isolated rat hepatocytes were exposed to CCl4 (1%) along with/without various concentrations of the total alkaloid fraction (80–40 µg/ml). Protection of human liver-derived HepG2 cells against CCl4-induced damage was determined by the MTT assay. Twenty-four healthy Wistar albino rats (150–200 g) of either sex were used for the *in vivo* investigations. Liver damage was induced by administration of 30% CCl4 suspended in olive oil (1 ml/kg body weight, i.p).

**Results:**

The antihepatotoxic effect of the total alkaloid fraction was observed in freshly isolated rat hepatocytes at very low concentrations (80–40 µg/ml). A dose-dependent increase in the percentage viability was observed when CCl4-exposed HepG2 cells were treated with different concentrations of the total alkaloid fraction. Its *in vivo* hepatoprotective effect at 80 mg/kg body weight was comparable with that of the standard Silymarin at 250 mg/kg body weight.

**Conclusion:**

The total alkaloid fraction was able to normalize the biochemical levels which were altered due to CCl4 intoxication.

## Introduction

*Hygrophila auriculata* (Acanthaceae) is an erect semi woody plant with annual growth up to 1.5 m in height.[1] In homeopathy, it is being used to treat jaundice, diseases of urogenital tract, arthritis, and constipation.[2] It is known for its anti-inflammatory and anti-oxidant activity.[2] Several plants belonging to the genus *Hygrophila* are known to possess liver protective effects[3] and steroidal alkaloids have been isolated from this plant.[4] However, the hepatoprotective effect of alkaloid fraction of the plant *H. auriculata* has not been determined. Hence, this study was intended to investigate the *in vitro* and *in vivo* hepatoprotective effects of the total alkaloid fraction of the leaves of *H. auriculata*.

## Materials and Methods

### Materials

All chemicals were obtained from SD Fine Chemicals, Mumbai. 3-(4,5-Dimethyl thiazol-2-yl)-2,5-diphenyl tetrazolium bromide (MTT), collagenase, insulin, dexamethasone, minimum essential medium (MEM), Ham's F12 medium, Silymarin (Standard), and antibiotics were purchased from Sigma Chemical Co., St. Loius, MO, USA. Ecoline diagnostic kits were purchased from E – Merck, India. The human liver-derived HepG2 cell line was obtained from National Centre for Cell Science, Pune, India.

### Plant Material

Mature leaves of *H. auriculata* were collected from the fields in and around mavelikara village, Kerala, in the month of May 2006. The plant was authenticated by Botanical Survey of India, Coimbatore, Tamil Nadu (Authentication no. BSI/SC/5/17/06-07/Tech 710).

### Preparation of the Plant Extract and Isolation of the Total Alkaloid

The fresh mature leaves (260 g) were subjected to a single extraction in a Soxhlet extractor using methanol (1 L) for 18–20 h. The extract was then concentrated to dryness under reduced pressure and controlled temperature to yield a dark green semisolid mass (18.8 g, 7.23%), which was preserved under refrigerated conditions. The total alkaloid fraction (yield 710 mg, 0.27%) was isolated from this extract using a conventional procedure.[5]

Thin-layer chromatography method was carried out on silica gel aluminum plate 60F-254, 0.5mm (TLC plates, Merck). The solvent system used for TLC was butanone/xylene/methanol/diethylamine (20: 10: 5: 1). The spots of both marker and extract were applied and the plates were developed and dried with help of a hair dryer. The spots were visualized by UV light at 254 nm, iodine vapor, and Dragendroff's spray reagent.

### Preparation of Suspensions

The total alkaloid fraction of *H. auriculata* was dissolved in DMSO and the volume was made up to 10 ml with Ham's F-12/MEM to obtain a stock solution of 1 mg/ml concentration and stored at –20°C prior to use. Further dilutions were made to obtain different concentrations ranging from 40 to 80 *µ*g/ml with respective media and used for *in vitro* investigations. A suspension of the standard Silymarin powder was also prepared (250 *µ*g/ml) in a similar manner. The total alkaloid fraction and the standard Silymarin powder were suspended in sodium CMC (0.3%) in distilled water separately and used for *in vivo* investigations.

### Hepatoprotective Effect of the Plant Extract in Freshly Isolated Rat Hepatocytes Isolation and culture of hepatocytes

Liver cells were isolated by a modified procedure of Seglen (1979).[6] Pentobarbital sodium (35 mg/kg b.w.) was used for anesthesia. Initially heparin was injected into the femoral vein (1000 IU) followed by perfusion with calcium free HEPES buffer 20 min (37°C), which contained 1% bovine serum albumin fraction V at a flow rate of 30 ml/min. The liver swells during this time, slowly changing its color from dark red to grayish white. The swollen liver was then perfused with a TPVG solution (50 ml) followed by perfusion with calcium-free HEPES buffer, which contained additional collagenase solution (0.075%) and calcium chloride (4mM) at a flow rate of 15 ml/min for 20 min.

After the perfusion, the lobes were removed and transferred into a sterile Petri dish containing calcium-free HEPES buffer and dispersed gently. It was transferred into a sterile conical flask and the crude cell suspension was stirred with the help of a magnetic stirrer for 5 min to release hepatocytes into the solution. The cell suspension was filtered through a nylon mesh (250*µ*) and the preparation was centrifuged at 1000 rpm for 15 min. The supernatant was aspirated and the loosely packed pellet of cells was gently re-suspended in the calcium-free HEPES buffer. This washing procedure was repeated three times. Cell viability was determined by the Trypan blue dye exclusion method.[7] The isolated hepatocytes were cultured in Ham's F12 medium, supplemented with 10% newborn calf serum, antibiotics, 10^-6^ M dexamethasone, and 10^-8^ bovine insulin, and the cell suspension was incubated at 37°C for 30 min in a humidified incubator under 5% CO_2_.

### Carbon tetrachloride-induced in vitro hepatocytes injury

Carbon tetrachloride (CCl_4_ )-induced hepatocytes injury was carried out. After an incubation of 24 h, the hepatocytes were exposed to the fresh medium containing CCl_4_ (1%) along with/without various concentrations of the total alkaloid fraction or the medium alone (as normal). Concentrations of the total alkaloid fraction greater than 80 *µ*g/ml and standard drug Silymarin greater than 250 *µ*g/ml were found to be toxic to the cells; hence, concentrations in the range of 40–80 *µ*g/ml were used for the total alkaloid fraction and 250 *µ*g/ml were used for the standard Silymarin. After 60 min of CCl_4_ challenge, concentrations of aspartate amino transferase (ASAT), alanine amino transferase (ALAT), alkaline phosphatase (ALP), triglycerides (TGL), total proteins, albumin, total bilirubin and lactate dehydogenase (LDH) in the medium were measured as an indication of hepatocytes necrosis using Ecoline diagnostic kits.[8]

### Hepatoprotective Effect in HepG2 Cell Line

The screening of hepatoprotective activity was based on the protection of human liver-derived HepG2 cells against CCl_4_-induced damage[9] determined by estimating mitochondrial synthesis using the tetrazolium assay.[10] The HepG2 cells were routinely grown and subcultured as monolayers in DMEM supplemented with 10% newborn calf serum. The experiments in this investigation were conducted with cells that had been initially batch cultured for 10 days. At this stage, the cells were harvested and plated at approximately 30,000 cells/well in 96 well-microtitre plates (Nunclon) and left to rest for 24 h at 37°C in a humidified atmosphere of 5% CO_2_. The cells were then exposed to toxicant (medium containing 1% CCl_4_ ) along with/without various concentrations of the total alkaloid fraction or the medium alone (as normal).[9] Concentrations of the total alkaloid fraction greater than 80 *µ*g/ml and standard drug Silymarin greater than 250 *µ*g/ml were found to be toxic to the cells; hence, concentrations in the range of 40–80 *µ*g/ml were used for the total alkaloid fraction and 250 *µ*g/ml were used for the standard Silymarin. At the end of the period, cytotoxicity was assessed by estimating the viability of the HepG2 cells by the MTT reduction assay.[10] After 1 h incubation, the test solution from each well was removed by aspiration and replaced with 50 *µ*l of MTT prepared in MEM without phenol red (MEM-PR). The plates were gently shaken and incubated for 3 h at 37°C in a humidified 5% CO_2_ atmosphere. The supernatant was removed and 50 *µ*l of propanol was added and the plates were gently shaken to solubilize the formed formazan. The absorbance was measured using a microplate reader at 540 nm.

### In Vivo Hepatoprotective Effect

Colony bred Wistar adult albino rats (150–200 g) of either sex were used for the investigations. All the animals were maintained under standard conditions with food and water *adlibitum*. The experimental procedures were approved by the Institutional Animal Ethics Committee (IAEC), KMC, Manipal (No. IAEC/KMC/06/2006-2007). The animals were divided into four groups of six animals in each group. Liver damage was induced by administration of 30% CCl_4_ suspended in olive oil (1 ml/kg body weight, i.p). Acute toxicity studies were performed and the dose was fixed at 80 mg/kg b.w. and 250 mg/kg body weight for alkaloid fraction and standard Silymarin, respectively. Group I received the vehicle (Sodium CMC 0.3%) and served as control and was not treated with the toxicant. The second group served as CCl_4_-treated control. Group III received a suspension of the total alkaloid of methanolic extract of leaves of *H. auriculata* (80 mg/kg b.w.), and group IV received the standard Silymarin (250mg/kg b.w.). The animals received these treatments by the oral route for a period of 7 days. On the seventh day except group I, all other groups received 30% CCl_4_ suspended in olive oil (1 ml/kg b.w.) i.p. After 24 h of intoxication, on the 8^th^ day, blood was collected in sterile centrifuge tubes and allowed to clot. Serum was separated and used for the estimation of ASAT, ALAT, ALP, TGL, total proteins, albumin, total bilirubin and LDH using Ecoline diagnostic kits.[8,11]

### Histopathological Examination

Liver was removed, fixed overnight in 10% buffered formalin, and paraffin-embedded. The sections were stained with hematoxylin and eosin (H and E) for histological evaluation and examined under light microscope. In brief, 4-*µ*m thick sections of paraffin-embedded mice liver were dewaxed in xylene, rehydrated in graded alcohol series, and washed with distilled water for 2 min. Subsequently, the sections were stained with hematoxylin for 5 min at room temperature. After 15 min, the sections were counterstained with eosin for 2 min, dehydrated in graded alcohol series, washed with xylene, and blocked by rosin. H and E- stained slides were observed under microscope at × 40 magnifications.

### Statistical Analysis

The statistical analysis was carried out by one way analysis of variance (ANOVA). The values are represented as mean ± S.E.M. Comparison of mean values of different groups treated with different dose levels of total alkaloid fraction and positive control with normal was performed by Turkey's Multiple Comparison Test. *P*< 0.05 was considered significant.

## Results

### Identification of Alkaloid Fraction by TLC

Thin-layer chromatography method was carried out on silica gel aluminum plate 60F-254, 0.5 mm (TLC plates, Merck). The solvent system used for TLC was butanone/xylene/methanol/diethylamine (20: 10: 5: 1). The chromatogram of marker compound showed single spot, where as the extract showed many additional spots with different R_f_ values. Among the several spots, one spot exactly matched with the marker compounds R_f_ value, and it was found to be more intense compared with the other spots. Hence, it indicated that the extract contains steroidal alkaloid.

### Hepatoprotective Effects in Freshly Isolated Rat Hepatocytes

The effects of the total alkaloid fraction of *H. auriculata* on freshly isolated rat hepatocytes intoxicated with CCl_4_ are recorded in Table 1. A significant increase in the levels of ASAT, ALAT, ALP, total bilirubin, LDH (*P* < 0.001) and a significant reduction in the levels of TGL, total proteins, and albumin (*P* < 0.001) were observed in hepatocytes exposed to CCl_4_ when compared to normal rats. These cells, when treated along with the total alkaloid fraction of *H. auriculata* showed a significant restoration of the altered biochemical parameters toward the normal (*P* < 0.001, when compared to the CCl_4_-treated group) and is dose dependent. A similar result was obtained when CCl_4_-intoxicated hepatocytes were treated with the standard Silymarin. However, the hepatoprotective effect of total alkaloid of *H. auriculata* was observed at very low concentrations (40–80 *µ*g/ml) when compared to the standard Silymarin. The decrease in the levels of ASAT, ALAT, total bilirubin, and LDH in freshly isolated hepatocytes treated with total alkaloid fraction at 80 *µ*g/ml was significant (*P* < 0.05 - 0.001, when compared to standard Silymarin) and more than that produced by the standard Silymarin at 250 *µ*g/ml.

**Table 1:** Effects of treatment of total alkaloid fraction of *Hygrophila auriculata* leaves on the biochemical parameters of CCl_4_ intoxicated freshly isolated rat hepatocytes

*Treatment*	*Concentration*	*ASAT U/L*	*ALAT U/L*	*ALP U/L*	*Albumin g/L*	*Total bilirubin mg/dL*	*Total protein g/dL*	*TGL mg/dL*	*LDH U/L*
Normal	-	15.00 ± 0.46	11 ± 0.39	34 ± 0.01	2.62 ± 0.06	0.20 ± 0.002	1.2 ± 0.04	162 ± 9.08	118 ± 0.005
CCl_4_	1%	84 ± 2.86^a^	58 ± 0.48^a^	98 ± 0.53^a^	0.9 ± 0.01^a^	0.68 ± 0.03^a^	0.4 ± 0.06^a^	71 ± 3.06^a^	298 ± 0.01^a^
CCl_4_ (1%) + standard silymarin	250 µg	18 ± 0.97^b^	14 ± 0.83^b^	36 ± 1.24^b^	2.14 ± 0.03^b^	0.28 ± 0.002^b^	1.14 ± 0.024^b^	156 ± 12.47^b^	128 ± 0.02^b^
CCl_4_ (1%) + total alkaloid fraction	80 µg	27 ± 0.98^b^	13 ± 0.92^b^	36 ± 1.22^b^	2.17 ± 0.04^b^	0.27 ± 0.002^b^	1.16 ± 0.02^b^	158 ± 10.08^b^	125 ± 0.04^b^
	70 µg	22 ± 1.39^b^	18 ± 0.73^b^	38 ± 0.66^b^	2.12 ± 0.07^b^	0. 28 ± 0.002^b^	1.13 ± 0.03^b^	155 ± 8.90^b^	127 ± 0.01^b^
	60 µg	25 ± 1.34^b^	20 ± 0.51^b^	39 ± 1.02^b^	2.08 ± 0.05^b^	0.30 ± 0.001^b^	1.10 ± 0.03^b^	151 ± 9.81^b^	131 ± 0.02^b^
	50 µg	28 ± 1.76^b^	24 ± 0.51^b^	42 ± 0.99^b^	1.99 ± 0.05^b^	0.32 ± 0.003^b^	1.07 ± 0.02^b^	149 ± 5.52^b^	134 ± 0.02^b^
	40 µg	29 ± 1.80^b^	27 ± 0.58^b^	44 ± 1.63^b^	1.94 ± 0.04^b^	0.34 ± 0.003^b^	1.02 ± 0.03^b^	146 ± 6.73^b^	137 ± 0.02^b^

Number of independent experiments (n= 3), 5 replicates, mean ± SEM;

a = *P* < 0.001, when compared to the normal group.

b = *P* < 0.001, when compared to the CCl_4_ group.

### Hepatoprotective Effects in the HepG2 Cell Line

The CCl_4_-exposed HepG2 cells showed a percentage viability of 17%. These exposed cells, when treated with different concentrations of the total alkaloid fraction of *H. auriculata*, showed a dose-dependent increase in percentage viability and the results were highly significant (*P* < 0.001, when compared to CCl_4_ intoxicated group). The percentage viability ranged between 78 and 92% at 80–40 *µ*g/ml concentration of the total alkaloid fraction [Table 2]. The increase in percentage viability of the HepG2 cells treated with total alkaloid fraction at 80 and 70 *µ*g/ml was significant (*P* < 0.01, when compared to standard Silymarin) and more potent than that produced by the standard Silymarin at 250 *µ*g/ml.

**Table 2:** Hepatoprotective activity of the total alkaloid fraction of *Hygrophila auriculata* leaves on CCl_4_ intoxicated HepG2 cells

*Treatment*	*Concentration (µg/ml)*	*% Viability*
Control	-	100
CCl_4_	-	17.65 ± 2.16^a^
CCl_4_ (1%) + standard silymarin	250	90.14 ± 3.12^b^
CCl_4_ (1%) + total alkaloid fraction	80	92.52 ± 4.21^b^
	70	90.15 ± 3.94^b^
	60	85.32 ± 3.65^b^
	50	80.43 ± 4.01^b^
	40	78.05 ± 3.03^b^

Average of 5 determinations (n=5), 4 replicates;

a = *P* < 0.001, when compared to the normal cells;

b = *P* < 0.01, when compared to the CCl_4_ intoxicated cells.

### In Vivo Hepatoprotective Effects

**Figure 1:**
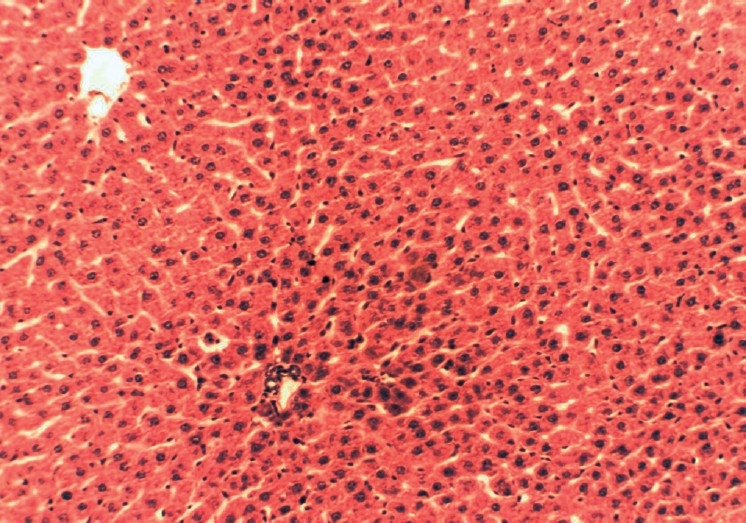
Histopathology of normal liver having normal histological structures of hepatic lobules

The effects of total alkaloid fraction of *H. auriculata* on CCl_4_-intoxicated rats showed that intoxication of rats treated with CCl_4_ significantly altered the biochemical parameters when compared with normal control rats (*P* < 0.001) [Table 3]. Treatment with total alkaloid fraction of *H. auriculata* at 80 mg/kg body weight showed a significant decrease in ASAT, ALAT, ALP, total bilirubin, LDH (*P* < 0.001) and a significant elevation in the TGL, total proteins, and albumin levels (*P* < 0.001) in serum when compared with CCl_4_-treated rats. Standard Silymarin at 250 mg/kg b.w. also exhibited similar results. All biochemical findings were positively supported by the histopathological results [Figures 1 and 4].

**Table 3:** Effects of treatment with the total alkaloid fraction of *Hygrophila auriculata* on the biochemical parameters of CCl^4^ intoxicated rats

*Treatment*	*Dose*	*ASAT U/L*	*ALAT U/L*	*ALP U/L*	*Albumin g/L*	*Total bilirubin mg/dL*	*Total protein g/dL*	*TGL mg/dL*	*LDH U/L*
Normal	-	89.48 ± 0.537	60.65 ± 13.55	328.37 ± 2.102	3.283 ± 0.295	0.46 ± 0.04	7.385 ± 3.127	76.57 ± 0.22	258.49 ± 0.051
CCl_4_	1ml/kg body wt	170.04 ± 2.04^a^	115.26 ± 12.18^a^	593.19 ± 4.604^a^	1.476 ± 0.104^a^	0.98 ± 0.02^a^	4.402 ± 1.42^a^	28.44 ± 0.12^a^	520.38 ± 0.04^a^
standard silymarin + CCl_4_ (1ml/kg body wt)	250 mg/kg body wt	80.87 ± 1.47^c^	62.15 ± 10.97^c^	358.32 ± 3.43^c^	3.374 ± 0.324^c^	0.44 ± 0.01^c^	6.459 ± 2.47^c^	68.02 ± 0.09^b^	305.72 ± 0.012^c^
total alkaloid fraction + CCl_4_ (1ml/kg body wt)	80 mg/kg body wt	86.92 ± 1.29^c^	64.55 ± 12.41^c^	370.09 ± 2.394^c^	3.368 ± 0.208^b^	0.48 ± 0.013^c^	6.202 ± 2.61^c^	67.08 ± 0.17^b^	312.59 ± 0.104^c^

Number of animals (n=6).

a = *P* < 0.001, when compared to the normal group.

b = *P* < 0.01.

c = *P* < 0.001, when compared to the CCl4 group.

**Figure 2:**
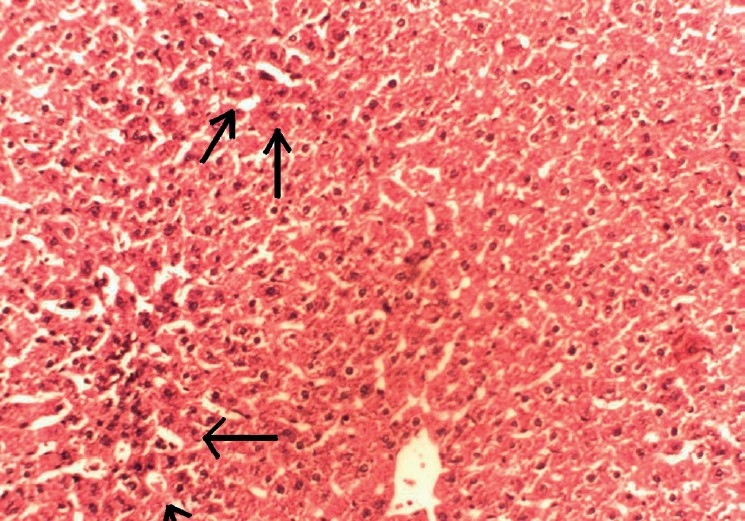
Histopathology of toxicant-treated liver (CCl_4_ 1 ml/kg body wt) showing damage to hepatocytes with hepatocellular vacuolization, focal hepatic necrosis, and congestion of hepatic sinusoids

**Figure 3:**
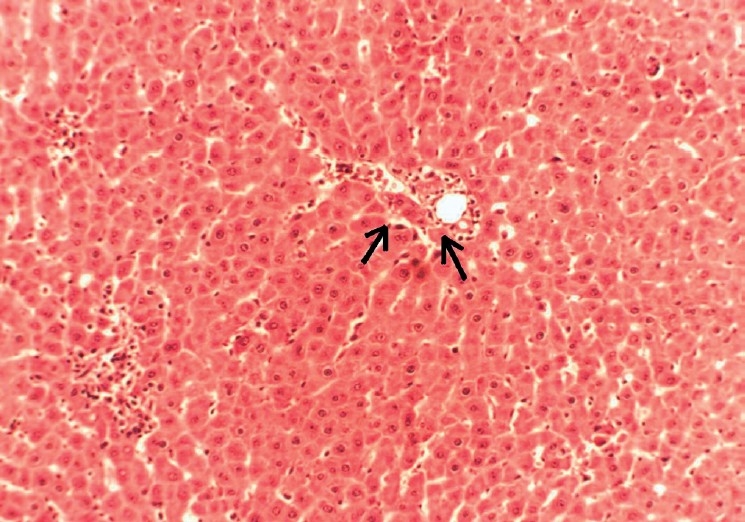
Histopathology of total alkaloid fraction of *Hygrophila auriculata* treated liver (80 mg/kg body wt) showing mild vacuolization

**Figure 4:**
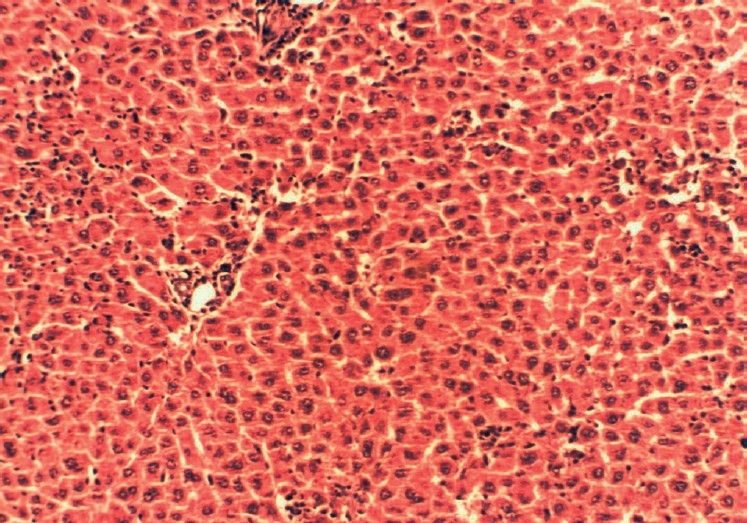
Histopathology of standard silymarin drug-treated liver (250 mg/kg body wt) showing apparently normal hepatocytes

## Discussion

Liver injuries induced by CCl_4_ are the best characterized system of xenobiotic-induced hepatotoxicity and commonly used models for the screening of anti-hepatotoxic and/or hepatoprotective activities of drugs.[12,13] Hepatic fibrosis was also investigated extensively.[14] Since the changes associated with CCl_4_-induced liver damage are similar to that of acute viral hepatitis,[15] CCl_4_-mediated hepatotoxicity was chosen as the experimental model.[16,17] It has been established that CCl_4_ is accumulated in hepatic parenchyma cells and metabolically activated by cytochrome P450-dependent monooxygenases to form a trichloromethyl radical (CCl_3_ ). The CCl_3_ radical alkylates cellular proteins and other macromolecules with simultaneous attack on polyunsaturated fatty acids, in the presence of oxygen, to produce lipid peroxides, leading to liver damage.[18] Thus, antioxidant or free radical generation inhibition is important in protection against CCl_4_-induced liver lesions.[19] Hepatotoxic compounds such as CCl_4_ are known to cause marked elevation in serum enzymes and bilirubin levels. It causes a marked decrease in total protein levels. Silymarin is used as standard hepatoprotective compound since it is reported to have a protective effect on the plasma membrane of hepatocytes.[20] To our knowledge, this is the first study which reveals the hepatoprotective effect of alkaloid fraction of *H. auriculata* against CCl_4_-induced toxicity in isolated rat hepatocytes, HepG2 cells in culture and in animal models. CCl_4_ has been found to induce extensive liver damage within a period of 24 h following intra-peritoneal administration. As a result of this, accumulation of fat in the liver and necrosis in the centrilobular region of the liver occurs. As a consequence, the microsomal enzyme activities are found to decrease and due to lipid peroxidation, the water-soluble enzymes leak into plasma from the liver. It is shown by the significant decrease in triglycerides and proteins in CCl_4_-intoxicated rat hepatocytes or animals in the present studies. Treatment with the total alkaloid fraction of *H. auriculata* exhibited significant restoration of the altered biochemical parameters toward normal in CCl_4_-intoxicated rat hepatocytes and in rats. Concentrations of the total alkaloid fraction greater than 80 *µ*g/ml and standard drug Silymarin greater than 250 *µ*g/ml were found to be toxic to the cells; hence, concentrations in the range of 40–80 *µ*g/ml were used for the total alkaloid fraction and 250 *µ*g/ml were used for the standard Silymarin. The effect of the total alkaloid at 80 *µ*g/ml was found to be better than that of standard Silymarin at 250 *µ*g/ml. Acute toxicity studies were performed and the animal dose was fixed at 80 mg/kg body weight and 250 mg/kg body weight for alkaloid fraction and standard Silymarin, respectively. Its hepatoprotective effect with *in vivo* studies at 80 mg/kg body weight was comparable to that of standard Silymarin at 250 mg/kg body weight, positively supported by the histopathology results.

Several plants belonging to the genus *Hygrophila* have also exhibited hepatoprotective effects. The aerial parts and the roots of *Hygrophila spinosa* (Acanthaceae) are used in herbal preparations.[3] The root of *H. spinosa* contains an alkaloid named hygrosterol.[4] *H. spinosa* has been investigated for its hematological parameters,[21,22] antitumor activity,[23] and its nutritional value.[24] An aqueous extract of *H. spinosa* 200 mg/kg body weight exhibited hepatoprotective activity against CCl_4_-induced liver injury in rats.[25] The antihepatotoxic effect of methanolic extracts of the seeds of *Apium graveolens* and *H. auriculata* was studied on rat liver damage induced by a single dose of paracetamol (3 g/kg p.o.) or thioacetamide (100 mg/kg, s.c.) by monitoring several liver function tests was first reported.[26] Treatment of diabetic rats with aerial parts of *H. auriculata* extract 100 and 250 mg/kg body weight for 3 weeks showed significant reduction in blood glucose, thiobarbituric acid reactive substances (TBARS). and hydroperoxide in both liver and kidney.[2] The hepatoprotective activity of the aqueous extract of the roots of *H. auriculata* was studied on CCl_4_-induced liver toxicity in rats.[27] Hence, the hepatoprotective effect observed in the present study may be mainly due to the presence of any of the steroidal alkaloids present in the total alkaloid fraction of the leaves of *H. auriculata*. The results from the present study indicate a good correlation between the *in vivo* and *in vitro* studies. In conclusion, the total alkaloid fraction merits further investigation to identify the active constituents responsible for this activity.
